# The effect of ginger (*Zingiber officinale*) feed on cardiac biomarker in medium-dose isoproterenol-induced myocardial toxicity

**Published:** 2021

**Authors:** Alaba Olumide Ojo, Omowumi Hilda Ekomaye, Oluwabukunmi Marvelous Owoade, Olatunbosun Olumuyiwa Onaseso, Lawrence Dayo Adedayo, Olufemi Idowu Oluranti, Emmanuel Olusegun Timothy, Abiodun Ayoka

**Affiliations:** 1 *Department of Physiology, College of Health Sciences, Bowen University, Iwo Nigeria*; 2 *Department of Physiological Sciences, Faculty of Basic Medical Sciences, Obafemi Awolowo University, Ile- Ife*

**Keywords:** Isoproterenol, Ginger, CK-MB, LDH, ALT, Cardiotoxicity

## Abstract

**Objective:**

Traditional medicines have been widely used to prevent and treat diseases for thousands of years. This study was designed to evaluate the effect of ginger feed on cardiac biomarker in isoproterenol (ISO)-induced myocardial toxicity.

**Materials and Methods:**

Thirty male Wistar rats were grouped into six groups of five: Control; ISO-induced toxicity; ginger fed; ginger fed before; ginger fed+ ISO simultaneously and ginger fed after. Freshly prepared solution of ISO was injected through intraperitoneal route at a dosage of 20 mg/kg, while the control received distilled water. Blood was collected via cardiac puncture after two weeks of administration, the serum was used to evaluate biomarkers.

**Results:**

The CK-MB and CK of ginger-fed groups were significantly lower compared to ISO group- 8.2±0.5 U/L and 39.36±5.28 U/L respectively, P <0.05. The CK-MB and CK levels of all ginger-fed groups showed no significant difference compared to the control- 2.2±0.3 U/L and 17. 07±3.4.90 U/L, respectively p>0.05, except ginger fed after group where they were significantly higher compared to the control. The mean value of LDH in all ginger-fed groups was lower than the ISO group (67.17±0.88 U/L; p<0.05), but significantly higher (p<0.05) than the control (26.45±2.52 U/L). The mean value of ALT in all ginger fed groups was lower than the ISO group (83.11±4.88U/L; p≤0.05).

**Conclusion:**

Ginger feed hindered toxic effects of isoproterenol.

## Introduction

Myocardial infarction is an acute condition of necrosis of the myocardium that occurs as a result of sudden or continual interruption of blood supply to the myocardium (Whellan, 2005). Myocardial infarction (MI) is a commonly spread manifestation of cardiovascular disease, and it is associated with an imbalance between coronary blood supply and myocardial demand (Awada et al., 2015). The incidence of myocardial damage could be due to hyperlipidemia, loss of plasma membrane integrity and membrane peroxidation (Krushna et al., 2009), and may occur due to coronary insufficiency leading to hypo-perfusion. Cardiovascular diseases was responsible for an estimated 17·8 million deaths in 2017 globally (Roth et al., 2018). By the year 2030, the United Nation Sustainable Development Goals aim to reduce premature mortality from non-communicable diseases by a third (UN. 2015).

ISO is a sympathomimetic drug that acts almost exclusively on beta-adrenergic receptors, used in the treatment of allergic emergencies, bronchial asthma, ventricular bradycardia, cardiac arrest, and glaucoma (Amano et al., 2007). However, ISO can induce side effects and cause some adverse reactions, which have been documented to produce MI in large doses (Long et al., 2012), as a result of cardiotoxicity.

Myocardial infarction induced by ISO has been reported to show many metabolic and morphologic aberrations in the heart tissue of the experimental animals similar to those observed in human myocardial infarction (Ithayarasi and Devi, 1997). There is also, an elevation of Ca^++^ overcharge inside the cardiomyocyte which is related to the activation of the adenylate cyclase enzyme and the depletion of ATP levels (Siddiqui et al., 2016). Evidence suggests that inflammation is a key process that mediates myocardial tissue damage after an ischemic event (Woudstra et al., 2017), Neutrophils infiltrate the infarcted area where they can promote myocardial cell damage via the release of proteolytic enzymes, inflammatory cytokines and chemokines, and the production of reactive oxygen species (Raish et al., 2017).

Traditional medicines and their preparations have been widely used to prevent and treat diseases for thousands of years in several countries (Long et al., 2012). The rise in the use of medicinal herbs and spices is as a result of the failing efficacy and toxicity related with conventional drugs and their inaccessibility to poor or low-income earners. Moreover, a number of studies have established the efficacy of traditional medicine for treating ischemic heart disease (Duan et al., 2017). 

Ginger *(Zingiber officinale*) belongs to the family of *Zingiberaceae*, and is cultivated in India and other parts of the world including Nigeria. From the ancient times, rhizomes of ginger have been consumed worldwide as a spice agent (Shukla and Singh, 2007) and has been reported as a widely used herbs in traditional medicine in many countries (Afzal et al., 2011). It is used in China as a digestive aid and remedy for nausea, and to treat disorders such as rheumatism and bleeding, baldness, snakebite, toothache and respiratory conditions (Duke and Ayensu, 1985). The principal components of the non-volatile part of ginger comprise of the polyphenolic compounds such as 6-gingerol, 6-shogaol, 8-gingerol, and 10- gingerol where 6-gingerol being the most abundant (Amran et al., 2005). The functional components of ginger have revealed many pharmacological properties, including cardio-protective, hepato-protective, anti-inflammatory, antioxidant, and antilipidemic activities (Ansari et al., 2006; Dugasani et al., 2010; Shanmugam et al., 2011). Both *in vitro* and *in-vivo* experiments showed that it possesses antioxidant actions in protecting against free radical damage that may activate the inflammatory response in the formation of atherosclerotic plaque, which is the precursor of most cardiovascular disease (Ahmed et al., 2000; Masuda et al.*,* 2004).

Previous studies on cardioprotective effect of ginger on experimentally induced damage in the heart, used ginger in extract form. However, there is scarce of scientific information on direct eating of ginger via feed on ISO-induced myocardial infarction in male Wistar rats. Therefore, the aim of this study was to evaluate the effect of ginger feed on cardiac biomarkers in ISO-induced myocardial dysfunction in male Wistar rats. We hypothesized that consumption of ginger may prevent myocardial infarction.

## Materials and Methods

**Experimental animals**

Thirty male Wistar rats weighing 140-200 g (8 to 12weeks old) were purchased from the animal house of the College of Health Sciences, Bowen University, Iwo, Osun State, Nigeria. The rats were fed with commercially available standard rat chow. The experimental procedures adopted in this study, were strictly in compliance with the Experimental Animal care and Use of Laboratory (NRC, 2011).

**Experimental design**

The animals were grouped into six groups of 5 rats each: Group I- (Control) was fed with normal feed and was not induced with ISO; Group II- (ISO) was fed with normal feed and induced with ISO; Group III- (Ginger) fed with ginger without ISO induction; Group IV- (Ginger before) ginger feed commenced 7 days before ISO administration began; Group V- (Ginger and ISO) fed with ginger feed and ISO induction simultaneously and Group VI- (Ginger after) ginger feed commenced 3 days after the commencement of ISO administration.

**Induction of myocardial infarction**

The solution of ISO was prepared by dissolving 250 mg of ISO in 25 ml of distilled water, each rat was injected 0.2 ml/100g via intraperitoneal route at a dosage of 20 mg per kg body weight. The dosage of ISO is in accordance with previous studies, seven days lasting ISO treatment (2 mg/kg) induced hypertrophy in the left ventricle (Fereira 2007), and medium doses of ISO (10–85 mg/kg) induced toxicity and structural changes (Nichtova et al., 2012; Zhang et al., 2007; Zhang et al., 2005; Rajadurai et al., 2007). The control was given distilled water at dosage of 0.2 ml/100g. 

**Preparation of ginger feed**

The ginger was washed and allowed to dry; then, it was cut into tiny slices and air dried to constant weight, then, it was grounded into powder. The rat feed and ginger were pelletized together, in a way that ginger constituted 10% of the feed.

**Organ and blood sample collection**

At the end of administration, the animals were sacrificed. Blood was collected through cardiac puncture into plain sample bottles. The blood samples were left to stand for 30 min to 1 hr then, centrifuged at 1000 rpm for 10 min. The serum was decanted and used for the evaluation of cardiac biomarkers (creatine kinase-MB, creatine kinase, lactate dehydrogenase and alanine aminotransferase). The heart was harvested and preserved in organ bottles containing 10% formalin solution for Heamatocylin & Eosin histological stain.

**Analysis of cardiac biomarker**

Creatine kinase-MB activity, creatine kinase, and alanine and aspartate aminotransferase (AST), were analyzed by a spectrophotometric method using appropriate kits (Apple, 1992; Adams et al., 1993; Glick et al., 1986; Kaplan and Pesce, 1989; Tietz, 1982; Committee, 1974).

**Histopathological studies**

The preparation of the slides involved steps in the methods described by (Bancroft and Gamble, 2008). The slides were viewed under Olympus microscope at magnification of X400 

**Statistical analysis**

Data were presented as mean±SEM and analysed using one way analysis of variance (ANOVA) and Tukey-Kramer Multiple Comparisons Test using SPSS-20.0. P values < 0.05 were considered to be significant

## Results

**Creatinine kinase myocardial band (CK-MB) levels**

**Figure 1:**
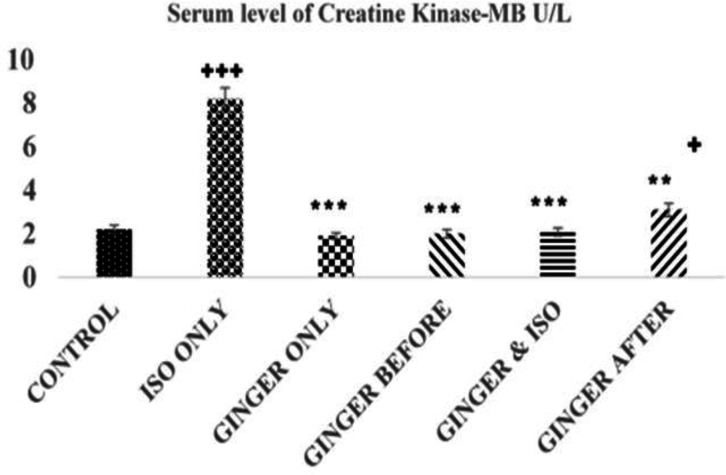
The serum levels of CK-MB (U/L) in each group. Values are expressed as mean±standard error of mean for the 5 animals in the group

The results of the CK-MB for the treated groups are shown in Figure 1. The results showed a significant decrease in serum CK-MB in the control and ginger fed groups compared to the ISO group (8.2±0.5 U/L, p < 0.05). There was no significant difference in serum level of CK-MB between ginger-treated groups compared to the control (2.2± 0.3 U/L, p>0.05) except ginger after group (3.1±0.5 U/L, p<0.05) that was significantly higher than the control. 

**Creatine kinase** **levels**

**Figure 2:**
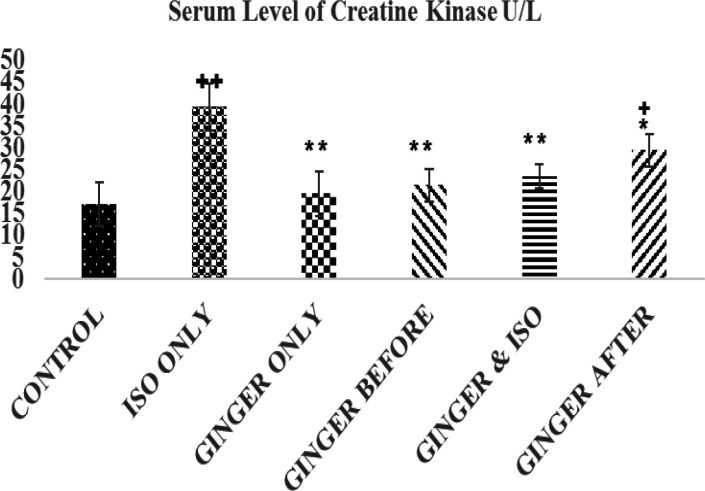
The serum levels of creatine kinase (U/L) in each group. Values are expressed as mean±standard error of mean for the 5 animals in the group

The results of the creatine kinase are shown in Figure 2. The result showed a significant decrease in serum creatine kinase in the control and ginger fed groups compared to the ISO group (39.36±5.28 U/L, p<0.01). There was no significant difference in serum level of creatine kinase between ginger treated groups and the control (17.06±4.91 U/L, p>0.05) except ginger after group (29.46±3.75U/L, p<0.05) that was significantly higher than the control group.

**Lactate dehydrogenase (LDH)** **levels**

**Figure 3:**
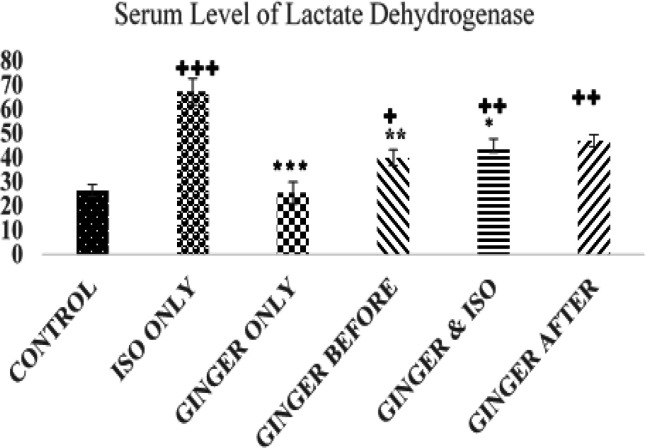
Chart showing the mean value of lactate dehydrogenase (LDH) in each group. Values are expressed as mean±standard error of mean. *=p<0.05, **=p<0.01, and ***=p<0.001 compared to the ISO group. +=p<0.05, ++=p<0.01, and +++=p<0.001 compared to the control group

The results of serum level of LDH are shown in Figure 3. The serum level of LDH in the control (26.45±2.52 U/L was significantly lower than the ISO group (67.17±2.5 U/L, p<0.001) and ginger treated groups except the ginger only group (25.41±5.53 U/L p>0.05). The serum level of LDH was significantly lower in ginger treated groups compared to the ISO group (p<0.05).

*=p<0.05, **=p<0.01, and ***=p<0.001 compared to the ISO group. +=p< 0.05, +++=p<0.001 compared to the control group.

*=p<0.05, and **= p<0.01, compared to the ISO group. +=p<0.05 and ++=p<0.01compared to the control group**.**

**Alanine aminotransferase** **levels**

**Figure 4:**
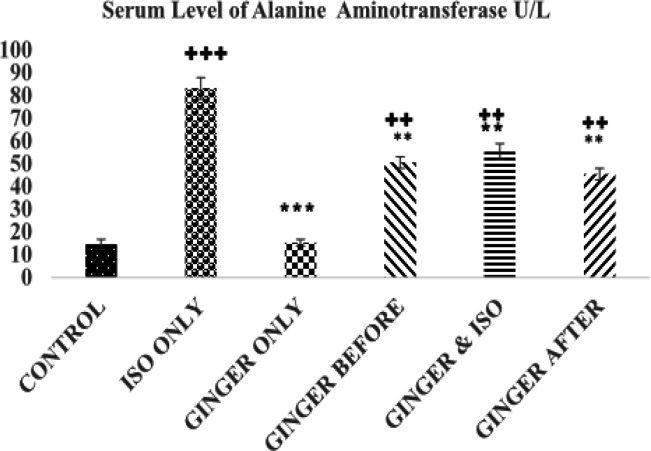
Chart showing the mean value of alanine aminotransferase (ALT) in each group. Values are expressed as mean±standard error of mean for the 5 animals in the group

The results of serum level of ALT are shown in Figure 4. The serum level of ALT in the control (14.53±2.22 U/L) was significantly lower than the ISO group (83.17±4.76 U/L, p<0.01) and ginger-fed groups except the ginger only group (15. 24±1.53U/L, p>0.05). The serum level of ALT was significantly lower in ginger-fed groups compared to the ISO group (83.17±4.76 U/L, p<0.01).

* =p<0.05, ** = p<0.01, and *** = p<0.001 compared to the ISO group. ++ =p<0.01, and +++ =p<0.001 compared to the control group.

**Histopathological results**

The histopathological results are shown in fig, 5 to 10 there was extensive myocardial necrosis in the ISO group compared to the control and ginger groups.

**Figure 5 (Group 1):**
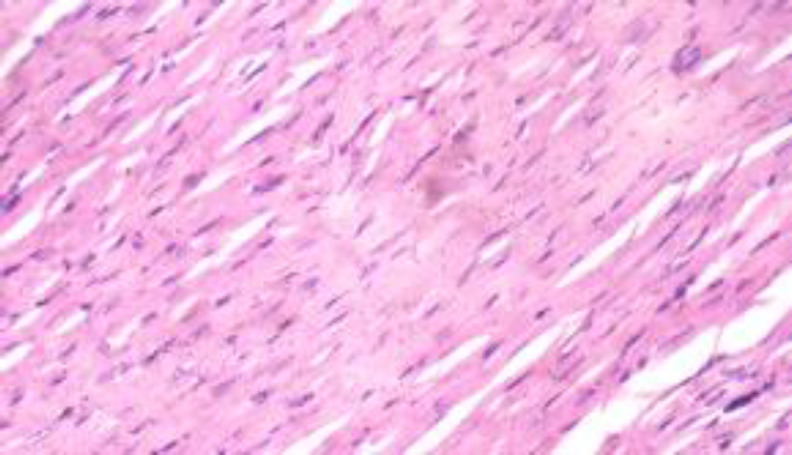
The cardiomyocytes appear normal. X 400

**Figure 6 (Group 2):**
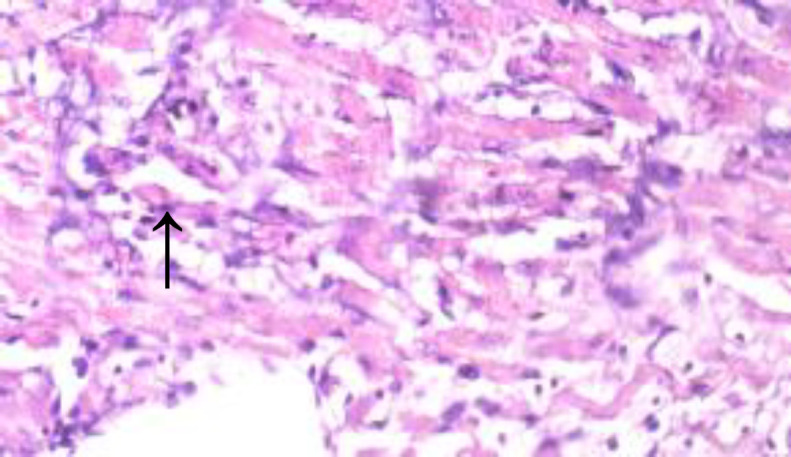
multiple foci of myofibre degeneration, fatty infiltration, necrosis, haemorrhage, and oedema X400

**Figure 7 (Group 3):**
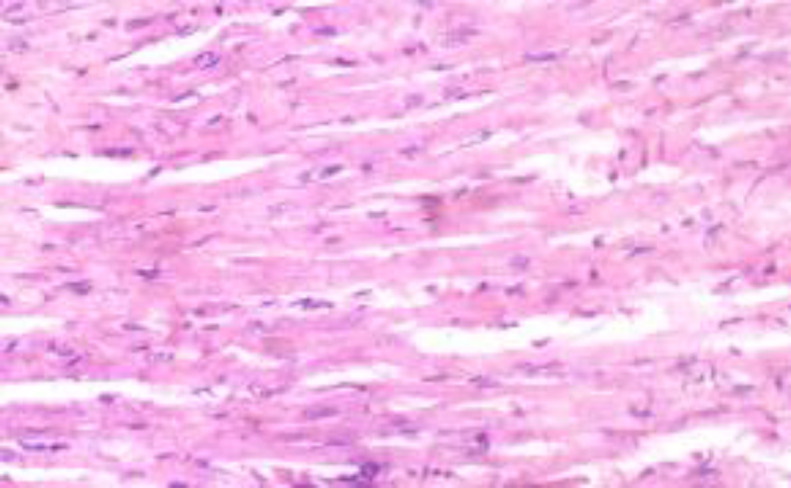
The cardiomyocytes appear fairly normal. There is no remarkable vascular change. X400

**Figure 8 (Group 4):**
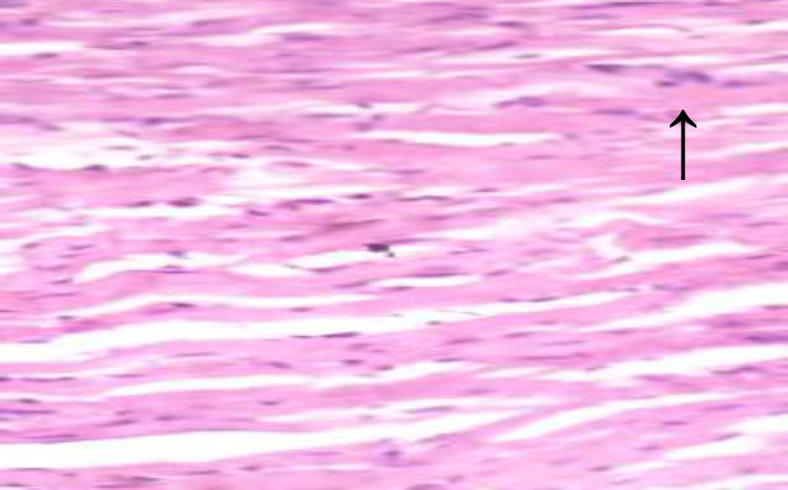
The cardiomyocytes appear fairly normal. There are however a few foci of mild degeneration of cardiac muscle fibres. X400

**Figure 9 (Group 5):**
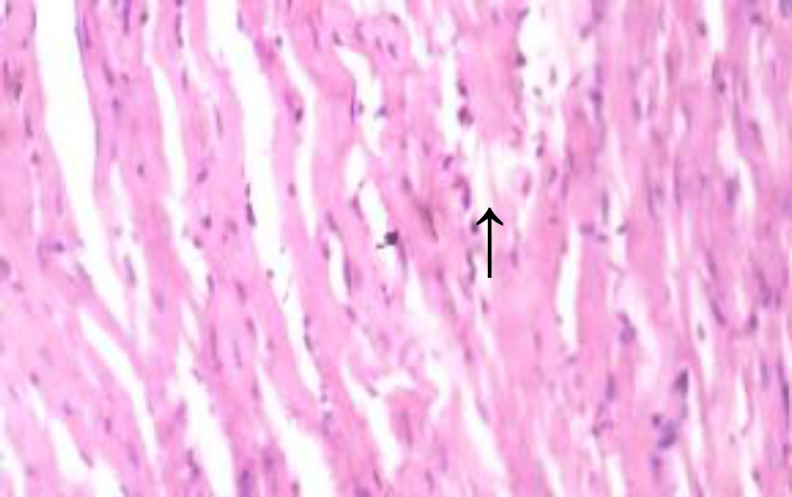
There are a few cellular infiltrate, oedema and haemorrhage in the sub-endocardial muscle bundle X400

**Figure 10 (Group 6):**
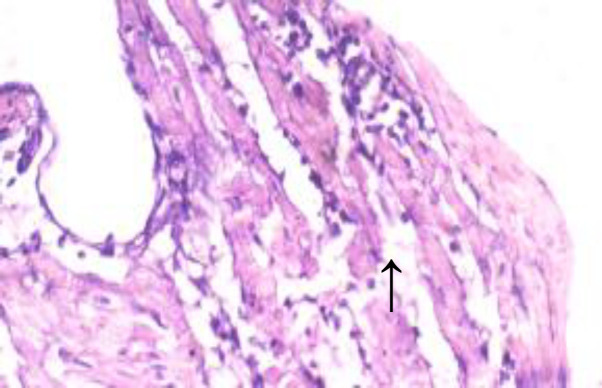
There is moderate atrophy, loss of myofibre striations and accentuation of the interstitium due to oedema X400

## Discussion

This study examined the effect of ginger feed on cardiac biomarkers in ISO-induced-cardiotoxicity in male Wistar rats. Intraperitoneal administration of ISO (20 mg/kg) resulted in an obvious toxicity as evidenced by marked sera elevations of creatine kinase (CK), lactate dehydrogenase (LDH), creatine kinase-myocardial band (CK-MB) and alanine aminotransferase (ALT) enzymes activities in the ISO group. Isoproterenol is a 𝛽-adrenergic agonist that has acute positive inotropic and chronotropic effects on the heart which can induce necrosis, inflammatory cell infiltration, hypertrophy, and fibrosis when administered at toxic dosage (Tasatargil et al., 2017). Several reports have shown extensive myocardial injury following ISO induction (Heraldo et al., 2011; Acikel et al., 2005; Ojha et al., 2011). When myocardial cells are damaged due to a deficiency in the oxygen supply or glucose, the cardiac membrane becomes permeable or may rupture entirely, resulting in the leakage of enzymes (Priscilla and Prince, 2009). Furthermore, the amount of the enzymes in serum is reported to be proportional to the extent of necrosis, which also reflects a nonspecific alteration in the plasma membrane integrity and/or permeability as a response to 𝛽-adrenergic stimulation (Saravanan et al., 2013). After myocardial ischemia, cardiomyocytes enzymes leak out from the injured tissues to the blood because the myocardium membrane becomes permeable due to deficient oxygen supply or glucose (Wand et al., 2018). 

This study showed that ISO injection led to a significant increase in CK-MB, CK, LDH and ALT activities (Figures 1-4). However, ginger fed rats: before, simultaneously and after ISO administration have remarkably lower levels of these biomarkers due to ISO toxicity as reflected by significant decreases in serum level of all the biomarkers analyzed in this study. These results corroborate an earlier report that ethanolic *Zingiber officinale *extract pre-treatment attenuated ISO-induced oxidative myocardial necrosis in rats (Ansari et al., 2006). The elevated level of myocardial cytosolic enzymes could be as a result of the increased generation of free radicals during ISO metabolism. These free radicals may be increased by calcium mediated proteases activities that could damage the cellular proteins and release cytosolic enzymes into the serum (Subbaiah et al., 2017). The property of ginger to reduce the level of CK-MB could be attributed to some of its constituents like 6-gingerol. The antioxidant activity of 6-gingerol has been proven to ameliorate the cardiac damage via reducing the free radical mediated lipid peroxidation and release of cytosolic enzymes from cardiac tissue (Mansour et al., 2008; Attyah et al., 2012).

The activity of CK, LDH and ALT enzymes are not present only in the myocardium, but are also found in profound quantities in the skeletal muscle, liver, and kidney, can reflect the degree of a tissue damage (Akila et al., 2017). ALT is majorly found in the liver; there is an increase release from the liver following hepatic ischemia secondary to cardiac dysfunction (Mcgrill, 2016). The increase in serum concentrations of ALT indicates cellular injury and inflammatory changes in the hepatic tissues (Peer et al., 2008). In this study, all these enzymes were higher in the ISO group than the control and the three groups fed with ginger. This supports the findings in previous studies that proved the tissue protective efficacy of ginger was manifested by its ability to reduce the circulating LDH and AST levels in rodents under oxidative stress conditions (Helal et al., 2012; Subbaiah et al., 2017). 

Combination of serum CK-MB, ALT and CK enzymes activities is important measures for both early and late phases of cardiac injury which might be due to the necrosis induced by ISO, though only CK-MB is the specific indicator of myocardial necrosis, because others are found in other tissues like the liver, kidney and skeletal muscle. The significant differences between biomarkers of ginger fed groups and ISO group reflect the cardioprotective potential of ginger feed.

The histopathology study revealed extensive myocardial necrosis in the ISO group while ginger-fed groups appeared fairly normal. The control group showed normal cardiac morphological structure. The ginger-fed before ISO appeared fairly normal than simultaneous and ginger after groups

The results of this study further corroborate the cardioprotective potential of ginger.
